# Pull-through technique through antegrade radial artery puncture without sheath insertion in balloon-assisted radiocephalic arteriovenous fistulas maturation

**DOI:** 10.1186/s12882-024-03523-z

**Published:** 2024-03-18

**Authors:** Jun Yin, Fengping Zhang, Ping Fu

**Affiliations:** 1 Department of Nephrology, Kidney Research Institute, West China Hospital of Sichuan University, Chengdu, China; 2 Department of Nephrology, Chengdu Second People’s Hospital, Sichuan Province, Chengdu, China; 3 Department of Nephrology, Jiujiang NO.1 People’s Hospital, Jiujiang, China

**Keywords:** Arteriovenous fistula, Pull-through technique, Hemodialysis, Balloon-assisted maturation

## Abstract

**Purpose:**

The aim of this study was to investigate the effectiveness and safety of the pull-through technique through antegrade radial artery puncture without sheath insertion in balloon-assisted radiocephalic AVF maturation.

**Methods:**

We retrospective studied a total of 62 patients with immature radiocephalic AVF, who received balloon-assisted maturation in our hospital. 15 patients received pull-through technique through radial artery without sheath insertion and 47 patients received treatment through a regular venous approach.

**Results:**

The success rate of pull-through technique group and control group was 86.7% (13 out of 15), 89.1% (41 out of 46) respectively. There was no significant difference between two groups (*P* > *0.05*). In our study, there were 2 patients in the pull-through technique group and 3 patients in the control group, which had hematoma in the vein puncture site (*P* = 0.59). There were also no differences in the primary patency rate between two groups at 6 months and 12 months (76.9% vs 70.7%, 38.4% vs 41.5%, respectively, *P* > 0.05).

**Conclusion:**

The pull-through technique through antegrade radial artery without sheath insertion in promoting radiocephalic AVF maturation is effective and safe.

## Introduction

Autogenous arteriovenous fistulas (AVFs) have been recognized as the primary long-term dialysis access for patients with end-stage renal failure, as recommended by the National Kidney Foundation Kidney Dialysis Outcomes Quality Initiative Clinical Practice Guidelines [1]. AVFs offer better patency, lower infection risk and lower healthcare cost in general population [2–4]. However, the initial failure rate, which was reported as high as 60% [5], bothered doctors and patients.

Currently, balloon-assisted maturation (BAM) technique is primarily employed to promote AVF maturation, because of the preservation of vascular resources [6]. However, when confronting the high-degree stenosis, multiple lesions and other difficult situations, a venous approach may require crossing difficult angles and kinks to access lesions. And it cannot be always succeeded. According to previous reports, antegrade artery puncture to introduce vascular sheaths can be used [7–9]. Although the reported complications resulting from trasnradial or transbrachial access are few [9, 10], it is also cannot be ignored that hematoma, occlusion or distal limb ischemia resulting from placement of sheath to the artery. Based on this, we have improved the technique by utilizing a pull-through (PT) method without insertion of sheath to address the issue. This study aims to compare the efficacy, safety, and 12-month primary patency rate between this technique and the conventional venous puncture retrograde access in promoting arteriovenous fistula maturation.

## Subjects and methods

### Participants

A retrospective study was conducted on patients received balloon-assisted maturation in our department between 2019 and 2020. A total of 65 patients received balloon-assisted maturation in our hospital, with one patient died from heart disease two months after intervention, and two patients receiving kidney transplantation within six months after intervention. Finally, there were 62 patients included in this study. 15 patients received balloon-assisted maturation with PT technique without sheath insertion through the antegrade radial artery approach. And 47 patients received a regular venous approach.

### Inclusion criteria

Patients who satisfied the following criteria were included in the study: (1) At least 8 weeks after creation of radiocephalic AVFs; (2) The range of stenosis was > 50% of the normal peripheral vessel diameter determined by ultrasonography; 3) the blood flow was < 500 mL/min; (4) AVF cannot provide 200 ml/min pump blood flow or it was too hard to puncture. (5) no signs of central venous disease.

### Intervention procedure

**Fig. 1:**
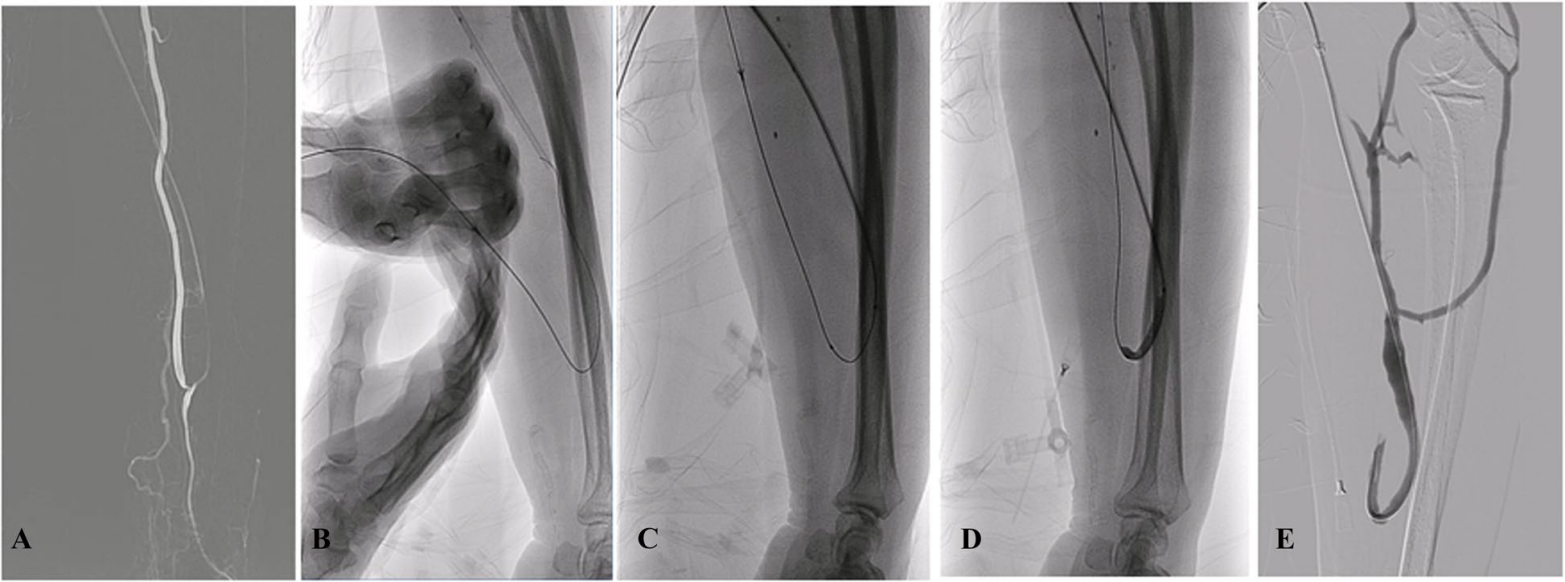
The intervention procedure of PT group. **A** Angiography shows high-degree stenosis of juxta-anastomotic segment. **B**,** C** antegrade radial artery puncture and insert a guide wire into sheath from the needle sleeve using pull-through technique. **D** Put the guide wire into brachial artery and dilate the stenosis. **E** Angiography to make sure there is no stenosis left

All patients underwent brachial plexus nerve block anesthesia before the start of the intervention. Once the anesthesia was deemed satisfactory, the Seldinger technique was used to insert a vascular sheath (5-6F, Terumo) via a retrograde vein puncture. A guidewire (RF*GA35153M, Terumo, Japan) and a Tempo Angiographic Catheter (451-514H0, Cordis Corporation, USA) were used to enter into the brachial artery through the anastomosis. Then angiography was performed to identify the precise site of stenosis, and an appropriately sized balloon (4-6 mm, Boston Scientific) was used for dilation. This was the standard procedure via a venous approach. If it was not possible to enter the proximal radial artery through the anastomosis, the ultrasound (VINNO 8, China) was used to guide antegrade radial puncture (the puncture needle was from the sheath introducer set, which allows a 0.035 inch guidewire to pass through its sleeve), which 3–5 cm proximal to the anastomosis. After successful puncture, the needle was removed, a guidewire (RF*GA35153M, Terumo, Japan) was inserted through the needle sleeve. After capturing the guidewire from the sheath by pull-through technique, the Catheter was sent to proximal radial artery. Then remove the guidewire and needle sleeve, local pressure was applied for 5 min. If no complications were found on ultrasound, the guidewire was re-inserted through the tempo angiographic catheter to the proximal radial artery and then it was sent to the brachial artery. Angiography was performed to identify the site of stenosis, followed by balloon dilation. This was the standard procedure for the PT group (Fig. 1).

### Post intervention follow up

All patients underwent evaluation of fistula maturation in our outpatient department 4–8 weeks after intervention. Subsequently, general and ultrasound examinations (VINNO 8, China) were conducted at least once every 3–6 months, and telephone follow-ups were performed every 6 months using a questionnaire (including blood pump flow, venous pressure, arterial pressures).

### Statistical analysis

Data are expressed as the mean ± SD or the percentage. Primary patency duration are expressed as the median and interquartile ranges. Continuous data were compared using Student’s t-test. A chi-square test or a Fisher’s test were used for analysis of categorical variables. Primary patency was displayed using a Kaplan–Meier survival curve and Cox regression model are used to estimate variable’s effect on the primary patency duration. A *P*-value less than 0.05 was considered to be statistically significant. The data were analyzed by RStudio for Windows (R version 4.0.2).

## Results

### Demographic and clinical characteristics of patients

A total of 62 patients were included in this study. The demographic and clinical characteristic of patients were summarized in Table 1. The PT group consisted of 15 patients with an average age of 57.07 ± 14.96 years old. Among them, 6 were female. The preoperative blood flow was measured at 259.73 ± 78.06 ml/min, while the postoperative blood flow was measured at 680.40 ± 159.12 ml/min. 11 patients had hypertension, and 9 patients had diabetes. The most common site of stenosis was juxta-anastomotic segment. The control group consisted of 47 patients with an average age of 62.34 ± 13.06 years old. Among them, 22 were female. The preoperative blood flow was measured at 258.44 ± 76.31 ml/min, while the postoperative blood flow was measured at 672.19 ± 143.84 ml/min. 31 patients had hypertension, and 33 patients had diabetes. The most common site of stenosis was mixed site. Except for stenosis site, there were no significant differences between PT group and Control group in age, sex, blood flow before or after operation, patency duration, hemoglobin, albumin, serum calcium, serum phosphorus, serum parathyroid hormone levels and comorbidities of diabetes and hypertension. Much more patients in PT group tend to have stenosis in the juxta-anastomotic segment, while more Control group patients have stenosis in mixed site (*P* < 0.001).

**Table 1:** Comparison of demographic characteristics between PT and control group

**Demographics**	**Overall**, *N* = 62	**Control Group**, *N* = 47	**PT Group**, *N* = 15	***p*-value**
**Age(years)**	61.06(13.61)	62.34(13.06)	57.07(14.96)	0.23
**Sex**				0.87
Female	28/62(45.16%)	22/47(46.81%)	6/15(40.00%)	
Male	34/62(54.84%)	25/47(53.19%)	9/15(60.00%)	
**Stenosis Site**				< 0.001
Forearm cephalic vein	9 (14.52%)	9 (19.15%)	0 (0.00%)	
Radial artery	9 (14.52%)	5 (10.64%)	4 (26.67%)	
Mixed site	28 (45.16%)	26 (55.32%)	2 (13.33%)	
JAS	16 (25.81%)	7 (14.89%)	9 (60.00%)	
**BBF**	258.44(76.31)	258.02(76.60)	259.73(78.06)	0.94
**ABF**	672.19(143.84)	669.57(140.37)	680.40(159.12)	0.82
**HBP**				0.83
No	20 (32.26%)	16 (34.04%)	4 (26.67%)	
Yes	42 (67.74%)	31 (65.96%)	11 (73.33%)	
**DM**				0.67
No	20 (32.26%)	14 (29.79%)	6 (40.00%)	
Yes	42 (67.74%)	33 (70.21%)	9 (60.00%)	
**Duration**	6.00(5.00,8.00)	6.0(5.00,8.00)	7.00(5.00,8.00)	0.74
**Hb**	98.97(15.34)	99.13(15.18)	98.47(16.37)	0.89
**Alb**	36.19(5.15)	36.38(4.83)	35.60(6.21)	0.66
**Ca**	2.08(0.18)	2.08(0.17)	2.06(0.20)	0.68
**P**	2.12(0.42)	2.14(0.40)	2.06(0.49)	0.57
**PTH**	533.11(166.38)	526.66(177.22)	553.33(129.79)	0.53

Abbreviations: *JAS* Juxta-anastomotic segment, *BBF* Blood flow before intervention, *ABF* Blood flow after intervention, *HBP* Hypertension, *DM* Diabetes mellitus, *Duration* the primary patency duration, *Hb* Hemoglobin, *Alb* Albumin, *Ca* Serum calcium, *P* Serum Phosphate, *PTH* Parathyroid hormone, Categorical variables were expressed as frequencies and percentages. The continuous variables of normal distribution were described by means and standard deviation. Primary patency duration are expressed as median and interquartile ranges

### Success rate, operation complications and time

There were 2 patients in the PT group and 5 patients in the control group still cannot be successfully used 8 weeks after intervention (*P* = 1.00). And 2 cases in PT group and 3 cases in control group had hematoma in the vein puncture site (*P* = 0.59). The average operation time of PT group was 48.9 ± 18.2 min, and that of the control group was 39.7 ± 20.4 min (*P* = 0.68, see the Table 2).

**Table 2:** Success rate, operation complications and time in two groups

	PT group	Control group	*P*-value
Success rate	13 (86.67%)	41 (89.1%)	1.00
Operation complications	2 (13.33%)	3 (6.38%)	0.59
Operation time	48.92 ± 18.21	39.75 ± 20.47	0.68

Abbreviations: *PT* Pull-through technique

### Post-intervention primary patency

**Fig. 2:**
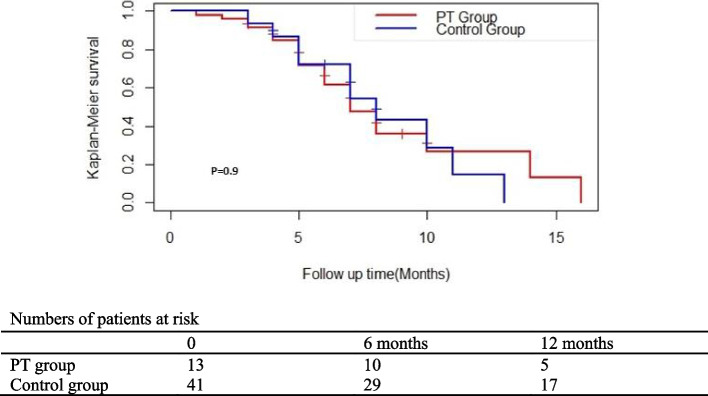
Comparison of the post-intervention primary patency rate between the PT and control groups

The median primary patency duration of PT group was 7 months (range from 3 to 13 months), while the control group was 6 months (range from 1 to 16 months). The post-intervention primary patency rate at 6 and 12 months in the PT group were 76.9% and 38.4%, and those in the control group were 70.7% and 41.5%. Figure 2 shows the Kaplan–Meier analysis for primary patency after intervention. There were no differences between the two groups at 6 and 12 months after intervention.

### Cox regression analysis for primary patency risk factors

We also conducted a univariate and multivariate Cox regression analysis to analyse clinical variables associated with primary patency. The result showed that the primary patency was not affected by the factors, including age, sex, stenosis site and group (See the Table 3).

**Table 3:** Cox regression of risk factors for primary patency duration

	Crude Model			Model1		
	HR	95%CI	*P*-value	HR	95%CI	*P*-value
Group						
Control group	Ref	Ref	Ref	Ref	Ref	Ref
PT group	0.94	0.52–1.69	0.8	0.74	0.35–1.55	0.40
Sex						
Female	Ref	Ref	Ref	Ref	Ref	Ref
Male	1.47	0.86–2.52	0.2	1.34	0.75–2.38	0.30
Age	0.99	0.97–1.01	0.2	0.98	0.96–1.00	0.11
Stenosis site						
Forearm cephalic vein	Ref	Ref	Ref	Ref	Ref	Ref
Radial artery	0.75	0.29–1.90	0.5	1.24	0.41–3.72	0.70
Mixed site	0.52	0.24–1.12	0.09	0.68	0.30–1.56	0.40
JAS	0.51	0.23–1.17	0.11	0.69	0.27–1.75	0.40

Model1: Adjusted for age, sex, stenosis site and group

Abbreviations: *JAS* Juxta-anastomotic segment, *HR* Hazard ratio *CI* Confidence Interval

## Discussion

The failure of arteriovenous fistula (AVF) maturation is one of the main problems encountered after AVF creation at present. In some situation, such as high-degree stenosis, juxta-anastomotic lesions and lesions at multiple sites of the AVF, transradial or transbrachial access for PTA with sheath placement has been proven effective [8, 10, 11]. In the professor Chee Wui Ong’s research, there were four patients with radial artery occlusion after sheath placement. Although most patients did not exhibit significant clinical manifestations postoperatively, there was still a concern about possible ischemia occurring in the distal extremities in the long run. In our study, we achieved satisfactory outcomes by employing pull-through technique using guide sleeve without sheath insertion for treating immature AVF. It not only reduces the risk of thrombosis, hematoma and pseudoaneurysm associated with sheath placement, but also reduces the complexity of the intervention to a certain degree.

In our follow-up of 62 individuals, we found the 6-month primary patency rates of the PT group and the control group was 76.9%, 70.7% respectively. The results were similar to those described in the professor Chen’s report [12]. Yet, at 12 months, the primary patency rate was much lower than previous results reported in the recent years [13]. This may be due to the fact that previous studies employed techniques like JXAS stenting, cannulation zone stenting and drug coated balloon angioplasty, while our research merely used plain balloon angioplasty. Additionally, during phone follow-up conversations for the reasons of re-intervention, we drew a possible conclusion that some patients chose to intervene earlier to avoid dialysis quality decreasing and thrombus formation. Because many of them received routine color Doppler ultrasound surveillance, which may reveal narrowed vessel diameter or decreased blood flow. This might explain why the 12-months primary patency rate in our study is on the lower side.

Previous studies have confirmed that the patency rate after PTA was influenced by several factors, such as stenosis characteristic and the drug-coated balloon use [5]. In our study, we employed the Cox regression model to analyze whether the route of entry affected the 12-month primary patency rate after invention. The results showed that there was no significant difference between the two groups, even after adjusted the factor, such as age, sex and stenosis site. The same conclusion was drawn upon the analysis of sex, age, and the stenosis site. In other words, it also demonstrated that the pull-through technique using guide sleeve without sheath insertion can also achieve comparable results in promoting radiocephalic AVF maturation.

In our study, all patients in the PT group underwent radial artery puncture guided by ultrasound, which could increase the success rate of puncture and avoid hematomas and occlusion. After successfully puncturing the radial artery, we used a regular 0.035-Inch guidewire to go through the needle sleeve. Once the guidewire was captured out from the sheath in the vein, the balloon could easier pass through the high-degree stenosis lesions or juxta-anastomotic segment. Besides, local pressure was applied for 5 min on the radial artery puncture site, without additional devices. This technique also reduced the medical cost. Meanwhile, it is also important to know that in case of juxta-anastomotic stenosis, the surgical technique could be a valid alternative to the endovascular technique if the venous approach does not allow to enter the proximal radial artery through the anastomosis.

In the follow-up of all included patients, we found that there were two patients int the PT group and 5 patients in the control group still cannot be used 8 weeks after intervention. They eventually chose the tunneled catheter as the dialysis access. All the other AVFs were successfully punctured and used 4–8 weeks after intervention, which confirming that the balloon-assisted maturation technique deem plays a significant role in promoting raidocephalic AVF maturation.

Similarly, when comparing the intervention time of both groups, we found that there was still no statistically difference. However, upon further investigation into all patients’ intervention time, we noticed that the operation time for early PT procedure patients was longer than those who underwent PT procedures later. That could be because, with experience gained from early PT techniques, the operator quickly switched to PT techniques when unable to pass through lesion sites during retrograde vein puncture. And it was also a meaningful measure for reducing radiation exposure time for both patients and operators.

## Conclusion

It is a comparably effective and safe method for promoting radiocephalic AVF maturation, especially when the guide wire cannot successfully pass through the anastomosis into brachial artery.

## Data Availability

The datasets generated and/or analysed during the current study are not publicly available due to the privacy of individual medical records but are available from the corresponding author on reasonable request.

## Abbreviations

AVF: Arteriovenous fistula
PT: Pull-through
BAM: Balloon-assisted maturation
